# The flushing procedure in nursing practices: A cross-sectional study with Portuguese and Brazilian nurses

**DOI:** 10.1016/j.heliyon.2020.e04579

**Published:** 2020-08-08

**Authors:** Pedro Parreira, Ricardo Vicente, Rafael A. Bernardes, Liliana B. Sousa, Beatriz Serambeque, Paulo Costa, Luciene M. Braga, Lisete Mónico, Anabela Salgueiro-Oliveira

**Affiliations:** a The Health Sciences Research Unit: Nursing (UICISA: E), Nursing School of Coimbra (ESEnfC), Coimbra, Portugal; b Department of Medicine and Nursing, Federal University of Viçosa, Viçosa, Minas Gerais, Brazil

**Keywords:** Health profession, Nursing, Health promotion, Health technology, Medical ethics, Peripherally intravenous catheter, Complications, Occlusion, Flushing

## Abstract

**Background:**

In patients with peripheral intravenous catheters (PIVCs), performing flushing is an essential procedure to maintain catheter patency and prevent complications. These PIVC related complications can lead to premature removal and therapeutics interruption, which implies the need of a new catheterization thus increasing patient discomfort and pain.

**Aims:**

To identify nursing practices related to the flushing procedure, namely: moment(s) of the flushing; the syringe size used; the flush solution, volume and technique; the knowledge and accomplishment of the recommended standards on flushing by nurses.

**Methods:**

A cross-sectional study was conducted between July and December 2017, with Brazilian and Portuguese nurses. An online questionnaire was developed based on the international recommendations on flushing procedure. Descriptive analysis was performed.

**Results:**

A total of 76 nurses answered the questionnaire. The majority of nurses (84.2%) performed flushing: the most common technique used was continuous syringe pressure (31.2%), with the push-pause technique being performed by 23.4% of the nurses. Despite the majority performs flushing at four distinct moments (after the PIVC insertion, before, between and after drug delivery), there are inconsistencies in flush solution, volume, and syringe size. The most used volume to perform flushing was 5 mL, filled using normal saline. Despite this, they also recognized the omission of this procedure due to time constrains, no familiarity with the procedure and unavailable material.

**Conclusions:**

This study identified that flushing procedure isn't always performed by nurses in their clinical practice. Also, several inconsistencies were observed between nurses that performed flushing, reflecting the lack of empirical evidence in this area of research.

## 1 Introduction

Peripheral intravenous catheters (PIVCs) are frequently used in hospital settings [1, 2] for a variety of purposes, but it often exposes persons to various risks, such as bloodstream infections, general discomfort, pain or swelling [2]. Catheter-associated bloodstream infections are also associated to higher morbidity and mortality risks [3, 4]. Additionally, some of the most frequent complications related to PIVCs are now well known in literature, as phlebitis [4, 5, 6, 7], infiltration [8] and occlusion [2, 4, 9, 10, 11], which often leads to catheter failure, intravenous therapy interruption, need for premature removal of PIVCs, being necessary a new catheterization [2, 12]. Occlusion is defined as any circumstance in which the PIVC does not enable to infuse fluids and is a clinical sign of catheter malfunctioning [1, 13, 14]. In fact, occlusion is often caused by fibrin coatings formed inside the PIVC lumen, which usually are built in the first 24 h of placement [1]. On the other hand, substance deposition inside the catheter increases the biofilm formation and the occurrence of several inflammatory or infectious complications [15]. Flushing the catheters with sodium chloride 0.9% is the most important practice in preventing malfunction by maintaining catheter patency [13]. The flushing technique is a common practice that should allow to maintain the patency of the catheter and reduce the risk of mixture of different substances by cleaning the inner lumen of the catheter [16, 17], also enabling the prevention of bacterial colonization of vascular access devices [4].

Although of extreme importance on reducing the risks and complications of PIVCs, this technique is complex and has a low evidence-based practice [18]. Nurses' practices appear to vary widely, regarding flush solutions and volumes, frequency, and techniques [13, 19, 20]. Flushing can be influenced by many factors like the healthcare professionals' education, which can influence their technique, but also the patient's venous system and associated comorbidities [13, 21]. Also, aspects like syringe diameter and injection flow dynamics are important components when applying flushing [13]. Despite the fact that it’s possible to find published international guidelines [16, 17, 22, 23, 24], it is also possible to find out that they vary widely, regarding recommendations on flushing moment, syringe volume, flush fluid and volume, technique and pressure used [20], which result in a lack of knowledge regarding the effects of the different followed protocols on PIVCs associated complications and risks*.* The current study has the purpose to identify nursing practices related to the flushing procedure, namely: moment(s) of the flushing procedure; the most common used syringe size; the flush solution, volume and technique; the knowledge and accomplishment of the recommended standards on flushing by nurses.

## 2 Methods

A cross-sectional study was conducted, using an online survey developed based on international recommendations and clinical expertise from the research team.

The recruitment was based on a convenience sample method, with the following inclusion criteria: being Portuguese or Brazilian nurses with at least one year of professional experience.

The link to the questionnaire was sent by e-mail to the University of Viçosa (Brazil) and to the Nursing School of Coimbra (Portugal), and then sent to each institution's collaborative network, between July and December 2017.

Regarding the questionnaire itself, it was composed by two main sections. The first part of the questionnaire was about professional experience in clinical practice, namely regarding flushing technique, moments performed, frequency, volume used, and other relevant data. The other section was a sociodemographic questionnaire, including a question about specific education in vascular access. Both sections had a total of 39 questions.

The questionnaire was composed of open and close questions about nursing practices on the flushing procedure, such as flushing moment, syringe volume, technique, and fluid quantity. Also, informed consent and sociodemographic data were collected from the participants.

An expert panel with five members – three PhD nurses and two MSc nurses - was created to develop the questionnaire, based on international guidelines. All the questions were evaluated by the same panel and were included only if there was 80% of agreement between experts, which allowed for a better validity.

The survey was approved by Ethic Commission (Brazil-CAAE: 99292918.8.0000.5153 and Portugal-P608-8/2019). All ethical considerations were strictly adhered to, namely the informed consent and voluntary participation, and legal aspects regarding privacy and confidentiality were also considered. Data were analysed using the Statistical Package for the Social Sciences – version 22.0 (SPSS 22.0, SPSS Inc., Chicago, IL, USA). Means, standard deviations, frequencies, and percentages were used as descriptive statistics.

## 3 Results

Although the questionnaire was answered by 309 nurses, only 76 questionnaires were considered for inclusion, since the others were not fully completed. From these, 64 professionals answered positively to the question about performing flushing. The study considered the 64 professionals, to describe the practices related to this procedure.

### 3.1 Sociodemographic data

The sample characterization is presented in Table 1.

**Table 1:** Sample characterization.

		N	%	M	SD
Sex	Male	19	25.0		
	Female	57	75.0		
Age (years)				33.4	7.1
Nationality	Portuguese	64	84.2		
	Brazilian Missing	11 1	14.7 1.3		
Education	Undergraduate	16	21.1		
	Post Graduate	31	40.8		
	Masters	26	34.2		
	PhD	3	3.9		
Working Place	Hospital	60	78.9		
	Primary or community	6	7.9		
	Other	10	13.2		
Vascular Access Training	Yes	62	81.5		
	No	14	18.5		

### 3.2 Flushing practices

From 76 questionnaires, 64 nurses (84.2%) recognized that they perform flushing during their clinical practice.

From the 12 nurses (15.8%) that mentioned the omission of the flushing practice 7 nurses (57.2%) refer that flushing isn't a common practice in their working place, 3 nurses (28.5%) state the lack of time to perform the technique, while 1 nurse (14.3%) recognizes material constrains, and 1 nurse (14.3%) states the non-familiarity with the flushing procedure. In fact, 29 nurses (38.2%) are not aware of the existence of specific guidelines about flushing in infusion therapy. From the nurses that know specific recommendations, most of them (83%) pointed the existence of specific internal institutional protocols.

### 3.3 Flushing moment

Regarding the moments of the flushing procedure, 52 nurses (78.8%) perform the flushing after PIVC insertion*,* 51 nurses (77.3%) flushes the PIVC before drug administration, 47 nurses (71.24%) perform the catheter flushing between drugs administrations*,* and 57 nurses (86.4%) flushes the PIVC after the last drug administration. The results revealed that the majority of nurses (47; 73.4%) perform the flushing on the four moments mentioned before.

### 3.4 Flushing technique

Despite the fact that 29 nurses (45.3%) didn't report the use of a specific technique, 35 nurses (54.7%) report such practice: 20 nurses (31.3%) use continuous syringe pressure and 15 (23.4%) use the *push-pause* technique.

### 3.5 Syringe size and volume used

Table 2 shows the main characteristics for flushing procedures in the four moments, specifically for syringe size, flush solution and volume used. Regarding the flush solution used, the majority uses manually prepared syringe with NaCl 0.9% in all the four moments considered in flushing procedure. Some nurses recognized the use of running infusion to perform flushing (7.7% after PIVC insertion, 11.8% before drug delivery, 12.8% between drug delivery and 14% after drug delivery). The pre-filled syringes with NaCl 0.9% are used by few nurses in their clinical practice (3.5% after drug administration; 6.4% between drug administration).

**Table 2:** Frequencies of reported flushing criteria (only for reported cases of Flushing).

		After PIVC insertion∗		Before drug delivery∗∗		Between drug delivery∗∗∗		After drug delivery∗∗∗∗	
		N	%	N	%	N	%	N	%
Flushing	Yes No	52 12	78,8% 18,2%	51 13	77,3% 19,7%	47 17	71,2% 25,8%	57 7	86,4% 10,6%
	TOTAL	64	100,0%	64	100,0%	64	100,0%	64	100,0%
Fluid	Running Solution	4	7,7%	6	11,8%	6	12,8%	8	14,0%
	Saline solution (NaCl 0,9%)	43	82,7%	39	76,5%	37	78,7%	44	77,2%
	NaCl 0,9% + Heparine	2	3,8%	3	5,9%	1	2,1%	3	5,3%
	Preffiled NaCl 0,9%	3	5,8%	3	5,9%	3	6,4%	2	3,5%
	TOTAL	52	100,0%	51	100,0%	47	100,0%	57	100,0%
Syringe Size	2 mL	8	15,4%	14	28,0%	12	26,1%	16	28,6%
	5 mL	30	57,7%	25	50,0%	22	47,8%	28	50,0%
	10 mL	10	19,2%	9	18,0%	10	21,7%	10	17,9%
	Other	4	7,7%	2	4,0%	2	4,3%	2	3,6%
	TOTAL	52	100,0%	50	100,0%	46	100,0%	56	100,0%
	missings			1		1		1	
Flush Volume	2 mL	14	27,5%	14	28,6%	12	27,3%	16	30,8%
	5 mL	23	45,1%	23	46,9%	21	47,7%	23	44,2%
	10 mL	7	13,7%	4	8,2%	5	11,4%	7	13,5%
	Other	7	13,7%	8	16,3%	6	13,6%	6	11,5%
	TOTAL	51	100,0%	49	100,0%	44	100,0%	52	100,0%
	missing	1		2		3		5	

The majority of nurses stated the use of 5mL syringes in all four moments (between 47.8% to 57.7%), followed by the 2mL syringes (from 15.4% after PIVC insertion to 28.6% after drug administration). In consonance with the syringes size used, the flush solution volume used most frequently in the four moments was 5mL (from 44.2% after drugs administration to 45.1% after PICV insertion), followed by 2mL, which is the second most common volume in the four moments.

## 4 Discussion

Flushing is an important procedure for the maintenance of PIVC and contributes to the prevention of some catheter-related complications, such as occlusion [13, 14, 15]. Taking into consideration the importance of the flushing procedure in reducing the PIVC related complications, this study shows a high percentage of nurses that perform flushing on their daily routine. Other cross-sectional surveys of nurses and midwives' practices reported a higher percentage of the respondents (91%) performing peripheral device flushing [22]. Around 38% didn't know specific standards, recommendations or guidelines on flushing procedures. Despite this, from the nurses aware of the existence of those guidelines, a higher percentage (83%) reported specific internal institutional protocols. In fact, some international guidelines highlight the importance of local policies and procedures that should be always adhered to [19, 25].

The flushing technique used is an important aspect for an efficient catheter cleaning [13]. Although having a small sample size, which is a meaningful limitation, it is possible to mention some important conclusions. In this study, 45.4% of the nurses reported that they do not use a specific flushing technique. From the nurses who use a specific technique to perform flushing, 31.3% use continuous syringe pressure and 23.4% use *push-pause* technique. In fact, international guidelines recommend the use of pulsatile technique and positive pressure [18, 19]. The guidelines specifically recommend positive pressure technique to minimize blood reflux into the PIVC lumen and ensure their adequate lock [18, 25]. This specific technique is also recommended if the patient is receiving intermittent injections or infusions, in order to promote and maintain patency and prevent the mixing of incompatible medications and solutions [25].

In this study, most nurses state that they perform flushing in all four different moments: after the PIVC insertion, before, between and after drug administration. These findings seem to be consistent with international recommendations for flushing procedure and also with previous studies on nursing practices relating flushing technique [26]. In fact, some guidelines clearly specify the need to flush the PIVC after their insertion to confirm their correct placement [25, 27], as well as before and after each drug administration [18, 19, 25, 26, 27]. Also, guidelines in general also recommend flushing between drug delivery, which is extremely important to reduce the risk of contact between incompatible medications [18, 19, 25, 26, 27].

Concerning the flushing solution used for the procedure, 78.8% of the nurses reported the use of the recommended normal saline solution (NaCl 0.9%). A previous cross-sectional survey [20] also identified the use of this solution for flushing procedure by the majority of the nursing and midwifery respondents (96%). The results of the present study are in accordance to the international guidelines [18, 19, 24, 25, 26, 27], which indicates that nurses accomplish with the international recommendations about flushing technique. In our study, few nurses use pre-filled syringes with NaCl, which might be an important topic for further research and educational support among nurses. In fact, some studies [28] reported that the use of pre-filled saline syringes are useful to reduce PIVC failure. Also, some nurses recognized the use of running infusion to perform flushing. According to previous studies [29], continuous infusing fluids has no influence on the appearance of PIVC-related complications. Although nurses often use this technique, the results of those studies highlight that this practice has no signifcant influence in PIVC duration, and also doesn't seem to reduce PIVC-related complications, thus having no efficacy for cleaning. In future studies, it would be necessary to examine its efficacy in the removal of deposits within the inner lumen.

Also, in this study, some of the nurses (between 7.7% to 14%) reported the use of a running continuous infusion to perform flushing, which is not aligned with the good practices for the flushing procedure [18, 19, 25, 26, 27]. In fact, there is evidence that this practice does not remove the content of catheter lumen adequately [30], if the infusion is not saline or has associated medications [13]. Also, glucose infusions usually used in running continuous infusions contribute to the build-up of glucose deposits, which also increases the risk for complications [13].

Regarding the syringe size, most nurses mentioned the use of a 5 mL syringe in the several moments. This finding is contradictory with the international guidelines that state the syringe size, namely 10mL [18, 19, 25, 26, 27] and bags of 50 mL [24] don't assure the eliminate all potential nesting material resulting in debris accumulation of sludge inside the PIVC [31, 32].

The survey about flushing practices in Queensland [22] identified the 10 mL syringes as the most used to perform the PIVC flushing, accomplishing with the international recommendations [18, 25, 26, 27]. In fact, the use of a 10 mL size syringe or larger is recommended in order to generate lower injection pressure [18] to avoid excessive pressure and blood vessels damage [19], thus avoiding the pressure of 25 psi [25]. In fact, depending on the syringe size, different pressures might be generated during flushing [33], and the syringe sizes need to be considered by the professionals in their clinical practice.

Concerning the volume of the flushing solution used, in consonance with the syringe size, nurses reported the use of 5 mL of flushing solution. The current international guidelines recommend that the volume used must be at least twice the volume of the catheter lumen [18, 19, 25]. Some guidelines indicate that the use of 5 mL for PIVC may remove more fibrin deposits, drug precipitation, and other debris [18]. Others refer to a minimum of 2 mL of flushing solution, considering the volume of catheters and add on devices [25]. However, nursing clinical judgment is crucial, and should take into consideration each particular context, specifically when involving additional volume from connectors and add-on devices, which may require more than 2 ml to ensure an adequate flushing. Despite this, it is also recommended to take into consideration the type and size of the catheter, patient's age, and type of infusion therapy being given [18, 19]. In fact, infusion of blood components, parenteral nutrition, contrast agents, and other viscous solutions may require larger flush volumes to a more effective cleaning of the catheter [13].

As other studies [12, 20, 34] already reported, the disparity in practices leads to the conclusion that it is imperative to motivate healthcare professionals for advanced training in vascular access and also raise the awareness of institutions, in order to create programs and periodic evaluations of their professionals. Thus, it is highly recommendable to engage nurses in these educational programs.

Farther to this, it is an advantage if, in the near future, healthcare schools enrich their curricula with the flushing dimensions referred before, also including specific advanced courses not only for professionals, but also for students.

## 5 Conclusions

Even considering the available literature about the need to use flushing in order to prevent risks and complications associated with PIVCs, this study identified that the procedure isn't always done and there are inconsistencies in nursing practices. This scenario may reflect the lack of guidelines consensus and lack of empirical evidence in this area of research. The several variables involved in the flushing technique, which were considered in this study (moment of the execution, technique, flush solution and volume, syringe size) justifies the complexity of this procedure. Still, nursing practices regarding those flushing variables could also be related with other factors such as PIVC material and gauge, drug viscosity, locking technique, velocity of drug administration, connectors and other add-on devices and their combined effect on the system. In fact, the generated pressure which is an important consequence of the combination of all mentioned variables is also a relevant issue.

This study had some limitations, especially the participants’ selection by convenience among the research team network and the small sample, which limits the generalizability and transferability of the results and their external validity. Future studies are needed about the efficacy of flushing on PIVCs cleaning according to the previous reported variables, preferably with greater sample sizes.

Additionally, we propose the development of a conceptual model including the variables reported before, to provide and achieve consistency in the current clinical practices. The research team already developed activities to contribute to this topic, namely the development of advanced courses in vascular access and the organization of an international congress in Portugal. Also, our Nursing School (Coimbra, Portugal), has invested in more theoretical and practical lectures about vascular access, specifically dedicated to the flushing technique, focusing on some dimensions that are being gathered to form a conceptual model, like *flushing moment, syringe volume, fluid used, flushing volume, catheter gauge, viscosity, administration velocity* and others. This model will contribute to the definition of important golden standards to better define best practices in PIVC maintenance.

## Declarations

### Author contribution statement

P. Parreira: Conceived and designed the experiments; Performed the experiments; Analyzed and interpreted the data.

A. Salgueiro-Oliveira: Conceived and designed the experiments.

L. Braga: Performed the experiments.

L. Mónico: Analyzed and interpreted the data.

L. Sousa, and R. Vicente: Analyzed and interpreted the data; Wrote the paper.

R. Bernardes, B. Serambeque and P. Costa: Contributed reagents, materials, analysis tools or data; Wrote the paper.

### Funding statement

This work was supported by the Research & Technological Development Incentive System (SI I&DT Co-Promotion; Project reference: POCI-01-0247-FEDER-017604).

### Competing interest statement

The authors declare no conflict of interest.

### Additional information

No additional information is available for this paper.
